# Supplementary material for: The DREAM complex functions as conserved master regulator of somatic DNA-repair capacities
Source: Nat Struct Mol Biol. 2023 Mar 23;30(4):475–88. doi: 10.1038/s41594-023-00942-8 (PMC10113156; doi:10.1038/s41594-023-00942-8)

nature structural & molecular biology

Article

https://doi.org/10.1038/s41594-023-00942-8

The DREAM complex functions as conserved master regulator of somatic DNA-repair capacities

In the format provided by the authors and unedited

Supplementary information Figure 1

a

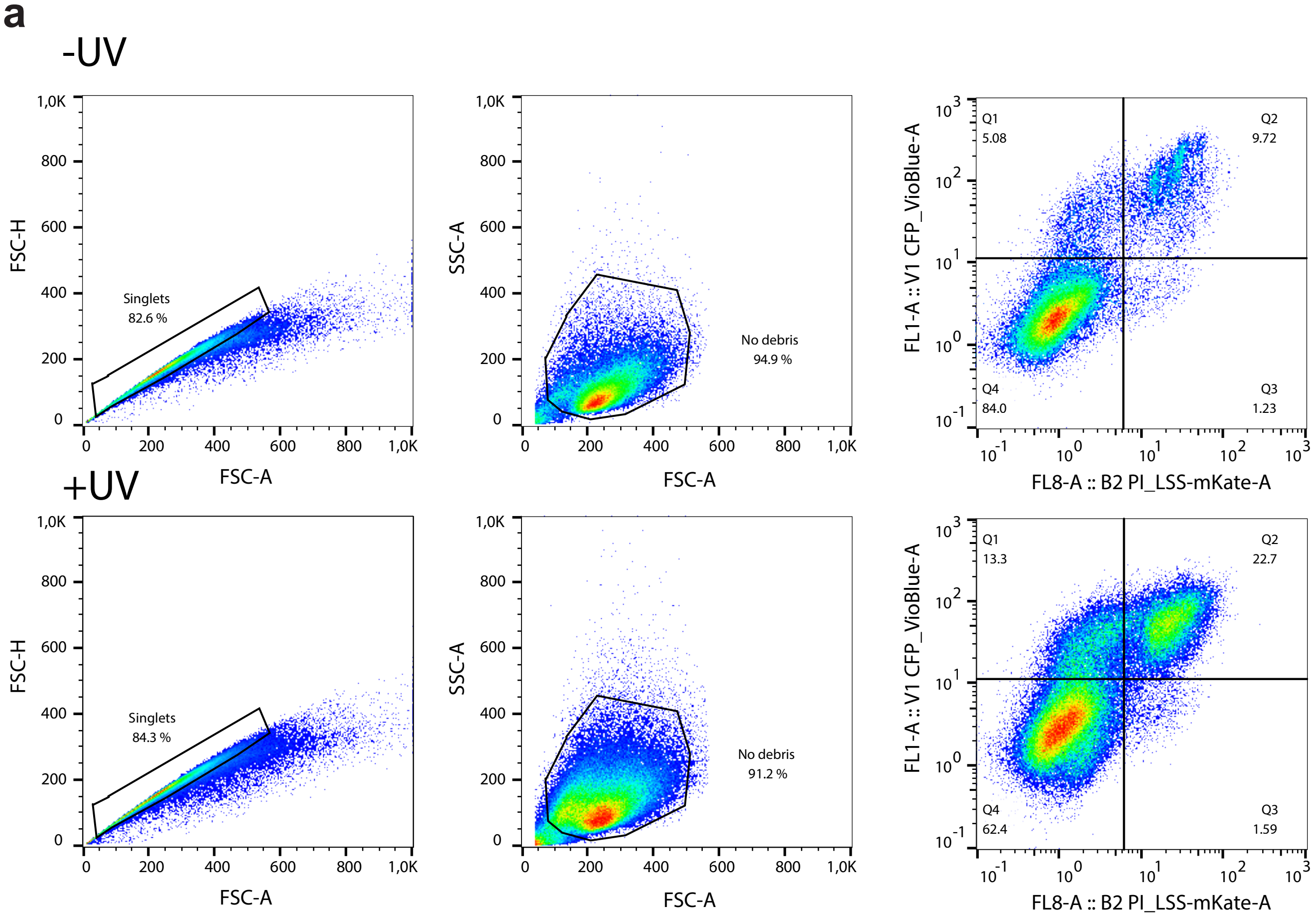

b

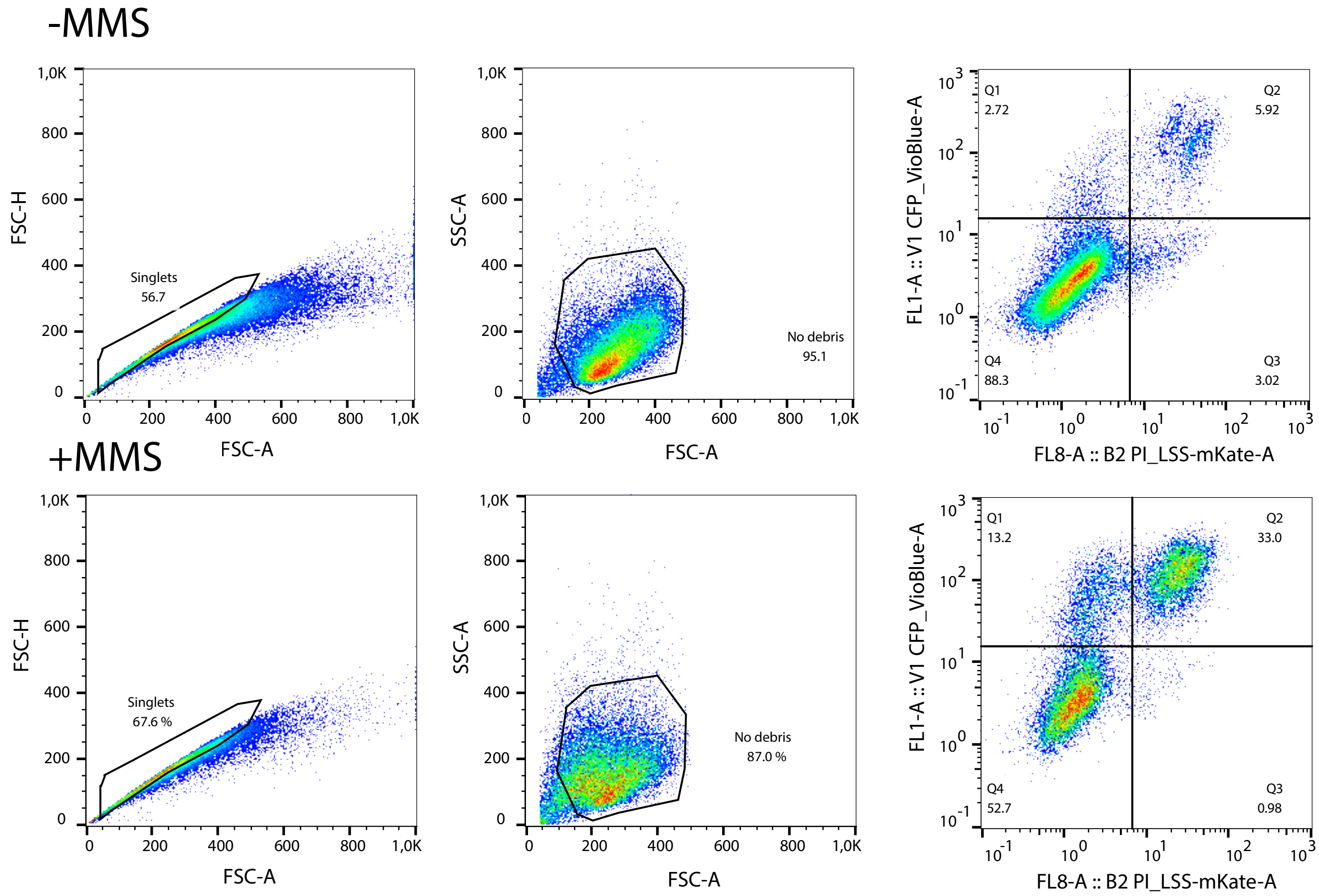

Supplement: Supplementary file 1 — Example images for the gating strategy used in flow-cytometry experiments for Fig. 1a, untreated and UV treated cells, and 1b, untreated and MMS treated cells. From left to right, singlets were selected using the FSC-H and FSC-A of the cells, followed by debris discrimination based on size and complexity (FSC-A and SSC-A), and finally, apoptotic cells were determined using the FL1 channel for annexin V (y axis) and FL8 channel for 7-AAD (x axis). [file 41594_2023_942_MOESM1_ESM.pdf]
