# Supplementary material for: The DREAM complex functions as conserved master regulator of somatic DNA-repair capacities
Source: Nat Struct Mol Biol. 2023 Mar 23;30(4):475–88. doi: 10.1038/s41594-023-00942-8 (PMC10113156; doi:10.1038/s41594-023-00942-8)
Supplement: Supplementary file 2 — Reporting Summary [file 41594_2023_942_MOESM2_ESM.pdf]

nature portfolio

Corresponding author(s): Björn Schumacher

Last updated by author(s): Feb 1, 2023

Reporting Summary

Nature Portfolio wishes to improve the reproducibility of the work that we publish. This form provides structure for consistency and transparency in reporting. For further information on Nature Portfolio policies, see our Editorial Policies and the Editorial Policy Checklist.

Statistics

For all statistical analyses, confirm that the following items are present in the figure legend, table legend, main text, or Methods section.

n/a	Confirmed	
☐	☒	The exact sample size (*n*) for each experimental group/condition, given as a discrete number and unit of measurement
☐	☒	A statement on whether measurements were taken from distinct samples or whether the same sample was measured repeatedly
☐	☒	The statistical test(s) used AND whether they are one- or two-sided<br>*Only common tests should be described solely by name; describe more complex techniques in the Methods section.*
☐	☒	A description of all covariates tested
☐	☒	A description of any assumptions or corrections, such as tests of normality and adjustment for multiple comparisons
☐	☒	A full description of the statistical parameters including central tendency (e.g. means) or other basic estimates (e.g. regression coefficient) AND variation (e.g. standard deviation) or associated estimates of uncertainty (e.g. confidence intervals)
☐	☒	For null hypothesis testing, the test statistic (e.g. *F*, *t*, *r*) with confidence intervals, effect sizes, degrees of freedom and *P* value noted<br>*Give P values as exact values whenever suitable.*
☒	☐	For Bayesian analysis, information on the choice of priors and Markov chain Monte Carlo settings
☒	☐	For hierarchical and complex designs, identification of the appropriate level for tests and full reporting of outcomes
☒	☐	Estimates of effect sizes (e.g. Cohen's *d*, Pearson's *r*), indicating how they were calculated

Our web collection on statistics for biologists contains articles on many of the points above.

Software and code

Policy information about availability of computer code

Data collection	qPCR CT values were obtained using Bio-Rad CFX Manager 3.0.<br>Videos for worm movement assessment were captured using ZEN 2.3 Pro software (Zeiss).<br>Other microscopy images were captured using LAS X 3.5.7 Life Science microscope software (Leica).<br>Flow Cytometry data was obtained using MACSQuantify software 2.13.0.
Data analysis	C. elegans RNA-seq data were processed through the QuickNGS pipeline, Ensembl version 85. The reads were mapped using Tophat (2.0.10) and abundance estimation was done with Cufflinks (version 2.1.1). DESeq2 was used for differential expression analysis.<br>Human RNA-seq data was processed with Salmon-1.1 and the output was summarized to the gene-level with tximport (1.14.2) and the differential gene analysis was done with edgeR (3.28.1).<br>C. elegans promoter and human promoter analysis was done using Homer's seq2profile function. p-values were calculated with the hypergeometric test function in scipy (1.5.1) and Python's statsmodels (0.11.1) was used to calculate the Benjamini-Hochberg FDR.<br>C. elegans proteomic data was analyzed by generating a prediced spectrum library using the Prosit webserver, and data was processed using DIA-NN 1.7.16 and imported to Perseus 1.5.5.0 for analysis.<br>Gene ontology analysis was performed using PANTHER 15.0 overrepresentation test.<br>Overlap analysis were done by using Fisher's exact test was done in R and gene set enrichment analysis (GSEA) was done in R v3.6.3 with the GSEA function of clusterProfiler v3.14.3. To calculate the adjusted p-values for the GSEA results, statsmodels v0.11.1 multipletests methods with the parameter method='fdr_bh' or method='bonferroni' in Python 3.6 was used.<br>2-tailed t-tests and the comparative Ct method for qPCRs were done using Microsoft Excel 2019.<br>Flow cytometry analysis was performed in FlowJo v10.7.1.<br>ANOVA of CPD signal data was performed with Python's pingouin v0.3.6.<br>Survival curves in C. elegans were analysed with Graphpad prism 7.03.

Microscopy image analysis was done using Imaris 9.9 (Oxford Instruments).
Venn Diagrams were done using Venn Diagram Plotter 1.5 and GIMP 2.10.12.
Motility of worms was analyzed using the plugin wrMTrck in ImageJ 1.53q.

For manuscripts utilizing custom algorithms or software that are central to the research but not yet described in published literature, software must be made available to editors and reviewers. We strongly encourage code deposition in a community repository (e.g. GitHub). See the Nature Portfolio guidelines for submitting code & software for further information.

Data

Policy information about availability of data

All manuscripts must include a data availability statement. This statement should provide the following information, where applicable:

- Accession codes, unique identifiers, or web links for publicly available datasets
- A description of any restrictions on data availability
- For clinical datasets or third party data, please ensure that the statement adheres to our policy

The C. elegans RNA-seq data used in this study are available from Gene Expression Omnibus (GEO; http://www.ncbi.nlm.nih.goz/geo) with the accession number GSE152235 and secure token yvqvewuetzkjrsl.
The C. elegans proteomics data used in this study is available via ProteomeXchange with identifier PXD033836.
The human RNA-seq data is available with the accession number GSE168401 and secure token wjehcacaflkxrip.
gencode-v37 transcripts and GRCh38.primary_assembly genome can be accessed from:
https://ftp.ebi.ac.uk/pub/databases/gencode/Gencode_human/release_37/
Ensembl version 85 data can be accessed from:
https://ftp.ensembl.org/pub/release-85/gtf/caenorhabditis_elegans/

Data from the following articles was re-analised, of which the availability is dependent on the journal and the institution from which it is accessed:
- Goetsch PD, Garrigues JM, Strome S. Loss of the Caenorhabditis elegans pocket protein LIN-35 reveals MuvB's innate function as the repressor of DREAM target genes. PLoS Genet. 2017 Nov 1;13(11):e1007088. doi: 10.1371/journal.pgen.1007088. PMID: 29091720; PMCID: PMC5683655.
- Reinke V, Gil IS, Ward S, Kazmer K. Genome-wide germline-enriched and sex-biased expression profiles in Caenorhabditis elegans. Development. 2004 Jan;131(2):311-23. doi: 10.1242/dev.00914. Epub 2003 Dec 10. PMID: 14668411.
- Tabuchi TM, Deplancke B, Osato N, Zhu LJ, Barrasa MI, Harrison MM, Horvitz HR, Walhout AJ, Hagstrom KA. Chromosome-biased binding and gene regulation by the Caenorhabditis elegans DRM complex. PLoS Genet. 2011 May;7(5):e1002074. doi: 10.1371/journal.pgen.1002074. Epub 2011 May 12. PMID: 21589891; PMCID: PMC3093354.
- Latorre I, Chesney MA, Garrigues JM, Stempor P, Appert A, Francesconi M, Strome S, Ahringer J. The DREAM complex promotes gene body H2A.Z for target repression. Genes Dev. 2015 Mar 1;29(5):495-500. doi: 10.1101/gad.255810.114. PMID: 25737279; PMCID: PMC4358402.
- Litovchick L, Sadasivam S, Florens L, Zhu X, Swanson SK, Velmurugan S, Chen R, Washburn MP, Liu XS, DeCaprio JA. Evolutionarily conserved multisubunit RBL2/p130 and E2F4 protein complex represses human cell cycle-dependent genes in quiescence. Mol Cell. 2007 May 25;26(4):539-51. doi: 10.1016/j.molcel.2007.04.015. PMID: 17531812.

Human research participants

Policy information about studies involving human research participants and Sex and Gender in Research.

Reporting on sex and gender	N/A
Population characteristics	N/A
Recruitment	N/A
Ethics oversight	N/A

Note that full information on the approval of the study protocol must also be provided in the manuscript.

Field-specific reporting

Please select the one below that is the best fit for your research. If you are not sure, read the appropriate sections before making your selection.

☒ Life sciences ☐ Behavioural & social sciences ☐ Ecological, evolutionary & environmental sciences

For a reference copy of the document with all sections, see nature.com/documents/nr-reporting-summary-flat.pdf

Life sciences study design

All studies must disclose on these points even when the disclosure is negative.

Sample size	Sample size are well stablished and commonly used on similar experiments in other scientific publications (Among others, Bianco et al, 2018, Rieckher et al 2017, Mueller et al 2014, Johnson NM et al 2013, Umansky et al, 2022)

Data exclusions	No data were excluded
Replication	In C. elegans, development growth assays and egg laying assays were performed a minimum of three independent times, each included 3 biological replicates per genotype and condition with an average around 40-50 individuals per sample and replicate across experiments. Slot blots were performed at least three independent times. Flow cytometry assays were done at least three independent times, each having 3 biological replicates within the experiment. Experiments from which single individual data was obtained, such as mice experiments, imaged worms, worms in lifespan assays, have the sample size indicated for each experiment. All attempts at replication were successful.<br>All performed experiments in mice are reported in the summary and all results were consistent.
Randomization	Randomization was not applied because the group allocation was guided based on the genotype of the respective mutant worms. Worms of a given genotype were nevertheless randomly selected from large strain populations for each experiment without any preconditioning. In mice experiments, allocation was random.
Blinding	Blinding was generally not applied as the experiments were carried out under highly standardized and predefined conditions such that an investigator-induced bias can be excluded. Developmental assays upon DNA damage with small observed effects were performed blinded to avoid any bias from the strain. This affects all replicates and repetitions of the developmental growth upon IR, MMS and Cisplatin.

Reporting for specific materials, systems and methods

We require information from authors about some types of materials, experimental systems and methods used in many studies. Here, indicate whether each material, system or method listed is relevant to your study. If you are not sure if a list item applies to your research, read the appropriate section before selecting a response.

Materials & experimental systems

n/a	Involved in the study
☐	☒ Antibodies
☐	☒ Eukaryotic cell lines
☒	☐ Palaeontology and archaeology
☐	☒ Animals and other organisms
☒	☐ Clinical data
☒	☐ Dual use research of concern

Methods

n/a	Involved in the study
☒	☐ ChIP-seq
☐	☒ Flow cytometry
☒	☐ MRI-based neuroimaging

Antibodies

Antibodies used	+ Primary antibodies used for immunofluorescence staining:<br>- Anti-CPD (Clone TDM-2, Supplier Cosmo Bio, CAC-NM-DND-001)<br>- Anti-6,4-PP (Clone 64M-2, Supplier Cosmo Bio, CAC-NM-DND-002)<br>- Anti phospho Histone H2A.X (Ser139) (Clone JBW301, Supplier Millipore, 05-636)<br>+ Secondary antibodies:<br>- AffiniPure Peroxidase-conjugated secondary antibody (Polyclonal, Jackson Immuno Research, 115-035-174)<br>- Anti-mouse IgG Alexa Fluor 555 (Polyclonal, Invitrogen, A-21422)<br>- Anti-mouse Alexa Fluor 488 (Polyclonal, Invitrogen, A-21202)
Validation	- Anti-CPD (Clone TDM-2, Supplier Cosmo Bio, CAC-NM-DND-001): https://www.cosmobiousa.com/products/anti-cpds-mab-clone-tdm-2<br>- Anti-6,4-PP (Clone 64M-2, Supplier Cosmo Bio, CAC-NM-DND-002): https://www.cosmobiousa.com/products/anti-6-4pps-mab-clone-64m-2<br>- Anti phospho Histone H2A.X (Ser139) (Clone JBW301, Supplier Millipore, 05-636): https://www.sigmaaldrich.com/DE/en/product/sigma/zrb05636?gclid=CjwKCAiApvebBhAvEiwAe7mHSK2Re-XHNNGZdzDlNOYV6VBEZ6fk6ECEIn2eEcMLrH0usRhY6n4WnhoC7aMQAvD_BwE&gclsrc=aw.ds<br>- AffiniPure Peroxidase-conjugated secondary antibody (Polyclonal, Jackson Immuno Research, 115-035-174): https://www.jacksonimmuno.com/catalog/products/115-035-174<br>- Anti-mouse IgG Alexa Fluor 555 (Polyclonal, Thermo Fisher Scientific, A-21422): https://www.thermofisher.com/antibody/product/Goat-anti-Mouse-IgG-H-L-Cross-Adsorbed-Secondary-Antibody-Polyclonal/A-21422

Eukaryotic cell lines

Policy information about cell lines and Sex and Gender in Research

Cell line source(s)	U2OS (cell line from tibia sarcoma of a female osteosarcoma patient) - ATCC Cat. No. HTB-96.
Authentication	Commercially available, visually and phenotypically as expected, no further authentication was performed.
Mycoplasma contamination	Regular mycoplasma testing showed no signs of contamination throughout the experiments.

Commonly misidentified lines (See ICLAC register)	No commonly misidentified lines were used.

Animals and other research organisms

Policy information about studies involving animals; ARRIVE guidelines recommended for reporting animal research, and Sex and Gender in Research

Laboratory animals	Species:<br>- Caenorhabditis elegans, strains are N2, MT8839, MT10430, MT15107, MT8879, MT11147, JJ1549, BJS634, RB951, RB1789, PFR40, MT8189, MT14390, RB1801, FX03886, RB864, FX04539, BJS21, BJS631, BJS629, BJS630, BJS772, BJS825, DW102, BJS890, BJS868, FX1524, BJS887, XF132, BJS722, hermaphrodites, ages ranging from day 1 to death (approx. 30 days maximum)<br>- Mus musculus, strain FVB/nj:C57BL/6j, age: 15 days old (postnatal)
Wild animals	No wild animals were used in this study
Reporting on sex	C. elegans were hermaphrodite for all experiments.<br>Mice:<br>For TUNEL assay, 3 WT (2M, 1F), 5 WT+H (3M, 2F), 7 ERCC1 KO (4M, 3F) and 7 ERCC1 KO+H (4M, 3F)<br>For γH2AX staining, 5 WT (3M, 2F), 5 WT+H (3M, 2F), 6 ERCC1 KO (3M, 3F) and 5 ERCC1 KO+H (3M, 2F)
Field-collected samples	No field-collected samples were used in this study
Ethics oversight	The animal facility at the Institute of Molecular Biology and Biotechnology (IMBB) operates in compliance with the "Animal Welfare Act" of the Greek government, using the "Guide for the Care and Use of Laboratory Animals" as its standard. Animal license 6ΛΤΑ7ΛΚ-ΚΚΘ issued by the Veterinary Medicine Directorate of Greek Republic.

Note that full information on the approval of the study protocol must also be provided in the manuscript.

Flow Cytometry

Plots

Confirm that:

☒ The axis labels state the marker and fluorochrome used (e.g. CD4-FITC).

☒ The axis scales are clearly visible. Include numbers along axes only for bottom left plot of group (a 'group' is an analysis of identical markers).

☒ All plots are contour plots with outliers or pseudocolor plots.

☒ A numerical value for number of cells or percentage (with statistics) is provided.

Methodology

Sample preparation	U2OS were cultured in DMEM, high glucose GlutaMAX Supplement, Pyruvate (Thermo Fisher Scientific, 31966047) with 10% fetal bovine serum (FBS; Biochrom GmbH, S0615) and 1% Penicillin-Streptomycin (Thermo Fisher Scientific, 15140112). Cells were kept at 37 °C in a 5% CO2 incubator (Binder). Cell dissociation from the plates was performed with Accutase (Sigma, A6964). To promote quiescence, cells were cultivated in FBS-free medium for 48 hours before genotoxic treatment were applied. 24 hours after FBS-free medium, cells were mock treated or received harmine hydrochloride (diluted in water) or INDY (diluted in DMSO) (Sigma, SMB00461 and SML1011) at 10 or 25 µM respectively. Before the genotoxic treatment, cells were washed with FBS-free medium. For the UV treatment, medium was removed from the plates and cells were irradiated using 254 nm UV-C light Phillips UV6 bulbs. The MMS treatment was performed by adding MMS at 2 mM for 2 hours, followed by 3 washes with FBS-free medium. Quantification via flow cytometry of cell death and apoptosis was performed 24 hours after genotoxic treatment. Collected cells were incubated in Annexin V Binding buffer (BioLegend, 422201) with Pacific Blue Annexin V (BioLegend, 640917) and 7-AAD (Thermo Fisher Scientific, 00699350) at 4 °C for 15 minutes. C
Instrument	Cells were measured using a MACSQuant VYB (Miltenyi Biotec).
Software	Cell data was obtained using MACSQuantify software 2.13.0, and analyzed using FlowJo (BD) v10.7.1.
Cell population abundance	Flow cytometry analysis was performed without sorting.
Gating strategy	Flow cytometry analysis started by selecting singlet events utilizing FSC-H vs FSC-A and selecting cells with a clear linear relation between height and area. FSC-A and SSC-A was utilized to separate debris, with low size and complexity, from the population of cells, which appeared clear. Finally, single cells were analyzed using the V1 channel (suitable for Pacific Blue, which is conjugated to the Annexin V) and the B2 channel (suitable for 7-AAD), as shown in the manuscript. The populations negative and positive for these markers were clearly defined, changed as expected upon DNA-damage-induced cell death and resembled similar experiments from the literature.

☒ Tick this box to confirm that a figure exemplifying the gating strategy is provided in the Supplementary Information.
