# Supplementary figures and images for: The DREAM complex functions as conserved master regulator of somatic DNA-repair capacities
Source: Nat Struct Mol Biol. 2023 Mar 23;30(4):475–88. doi: 10.1038/s41594-023-00942-8 (PMC10113156; doi:10.1038/s41594-023-00942-8)

# Anti CPD

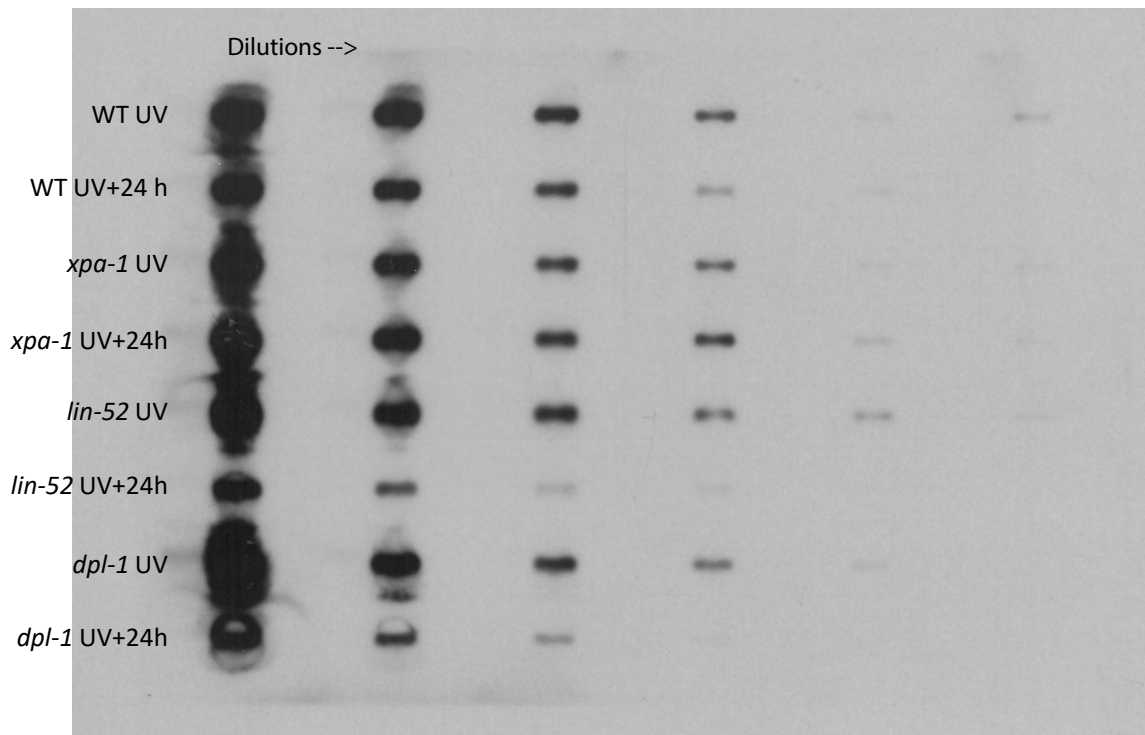

# DNA

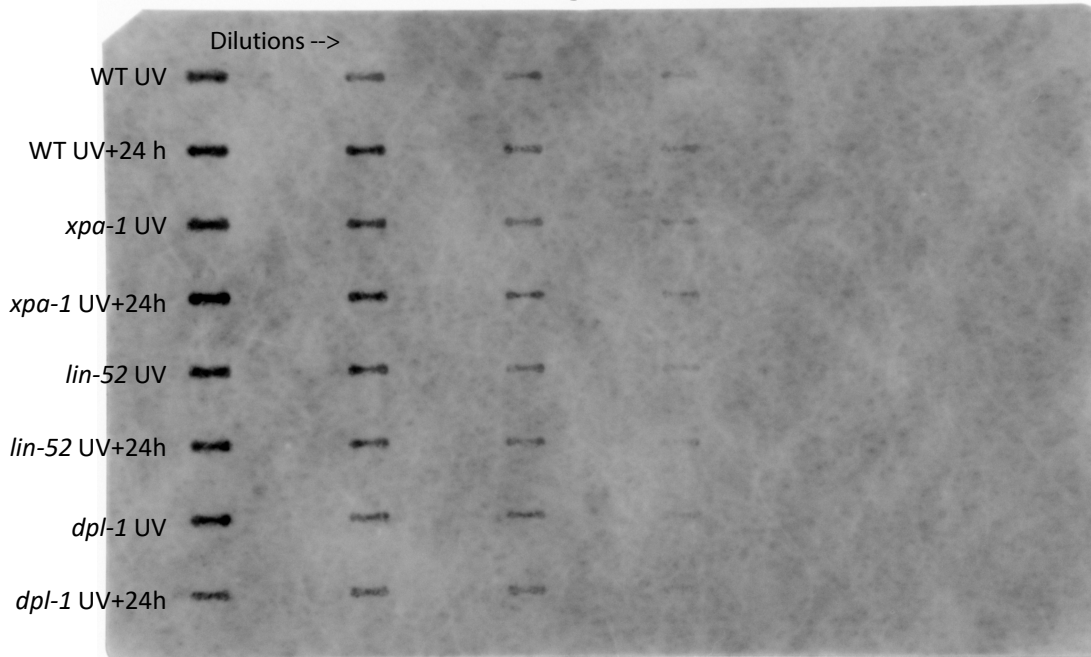

Supplement: Source Data Fig. 2 — Uncropped slot blot membrane. [file 41594_2023_942_MOESM5_ESM.pdf]

# Anti 6-4PP

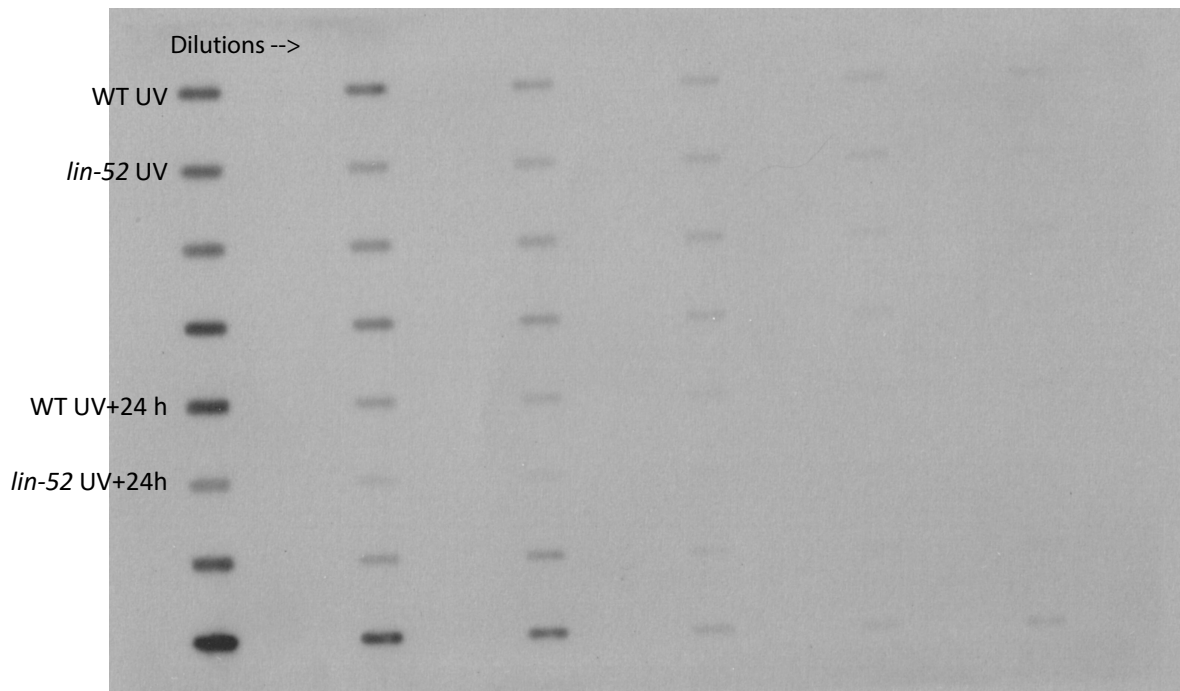

# DNA

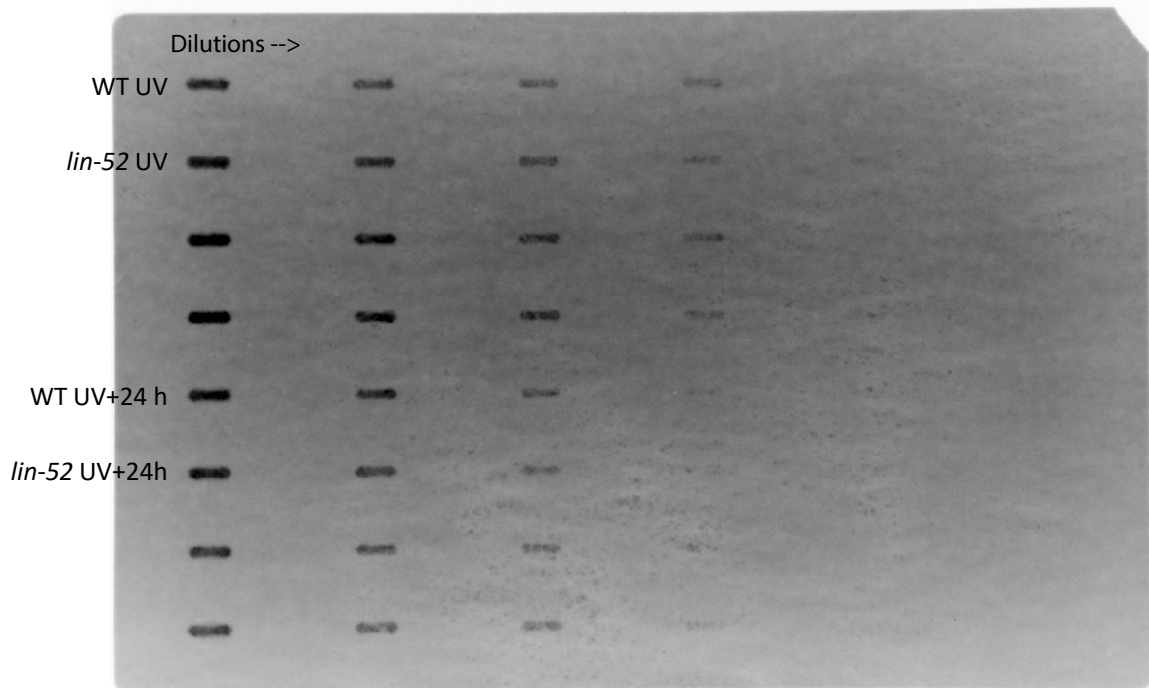

Unlabelled rows correspond to other samples not used in this study

Supplement: Source Data Extended Data Fig. 4 — Uncropped slot blot membrane. [file 41594_2023_942_MOESM6_ESM.pdf]
